# Marker-Trait Associations for Enhancing Agronomic Performance, Disease Resistance, and Grain Quality in Synthetic and Bread Wheat Accessions in Western Siberia

**DOI:** 10.1534/g3.119.400811

**Published:** 2019-10-23

**Authors:** Madhav Bhatta, Vladimir Shamanin, Sergey Shepelev, P. Stephen Baenziger, Violetta Pozherukova, Inna Pototskaya, Alexey Morgounov

**Affiliations:** * Department of Agronomy, University of Wisconsin-Madison, Madison, WI 53706,; † Omsk State Agrarian University, Omsk, Russia,; ‡ Department of Agronomy and Horticulture, University of Nebraska, Lincoln, NE 68583, and; § International Maize and Wheat Improvement Center (CIMMYT), Ankara, Turkey

**Keywords:** grain yield and yield-related traits, wheat rust, grain protein, gluten content, multiple traits, genome-wide association study, SNP markers

## Abstract

Exploiting genetically diverse lines to identify genes for improving crop performance is needed to ensure global food security. A genome-wide association study (GWAS) was conducted using 46,268 SNP markers on a diverse panel of 143 hexaploid bread and synthetic wheat to identify potential genes/genomic regions controlling agronomic performance (yield and 26 yield-related traits), disease resistance, and grain quality traits. From phenotypic evaluation, we found large genetic variation among the 35 traits and recommended five lines having a high yield, better quality, and multiple disease resistance for direct use in a breeding program. From a GWAS, we identified a total of 243 significant marker-trait associations (MTAs) for 35 traits that explained up to 25% of the phenotypic variance. Of these, 120 MTAs have not been reported in the literature and are potentially novel MTAs. In silico gene annotation analysis identified 116 MTAs within genes and of which, 21 MTAs were annotated as a missense variant. Furthermore, we were able to identify 23 co-located multi-trait MTAs that were also phenotypically correlated to each other, showing the possibility of simultaneous improvement of these traits. Additionally, most of the co-located MTAs were within genes. We have provided genomic fingerprinting for significant markers with favorable and unfavorable alleles in the diverse set of lines for developing elite breeding lines from useful trait-integration. The results from this study provided a further understanding of genetically complex traits and would facilitate the use of diverse wheat accessions for improving multiple traits in an elite wheat breeding program.

The spring wheat (*Triticum aestivum* L.) production region of Northern Kazakhstan and Western Siberia is approximately 15 million ha, in a belt 600-1000 km wide between the desert of Central Kazakhstan and boreal forests of Siberia (latitude 45-47° north). The crop is grown under continental climatic conditions with long cold winters, short hot summers, and annual rainfall that varies from 280-300 mm in the south to 400-450 mm in the north. Spring wheat is normally planted in mid-May and harvested in mid-September. A detailed description of the production environments, biotic and abiotic stresses, varieties, and breeding challenges was reported previously (Morgounov *et al.* 2007, 2008, 2010, 2013). Limited inputs and lack of irrigation result in low average yields of 1.2-1.5 t/ha, but cultivation is profitable for the farmers due to low growing costs and relatively high bread-making quality, which is valued in the market (Morgounov *et al.* 2008).

Both Kazakhstan and Russia are major wheat producers, self-sufficient in wheat grain (http://faostat.fao.org/), and increasingly ambitious in occupying the export markets. Unlike, modern cultivars grown in similar environments of North America, the spring wheat varieties grown in this region are tall and sensitive to day length (Trethowan *et al.* 2006). Even without the introduction of *Rht* (reduced height) and *Ppd* (photo period sensitivity) genes, or the 1B.1R translocation, Siberian varieties have achieved annual genetic gains of 0.7% since 1900 (Morgounov *et al.* 2010). In the late 1990s, CIMMYT began a collaborative research and breeding program with Northern Kazakhstan and Western Siberia, and by 2000 this had evolved into the Kazakhstan-Siberia Network for Spring Wheat Improvement (KASIB), comprising 18 breeding and research programs. The network focuses on two major activities: KASIB-YT (multi-location cooperative yield trials to evaluate disease resistance, grain yield, quality, and other traits) and shuttle breeding between the region and CIMMYT to incorporate stem rust (incited by *Puccinia graminis* f.sp. *tritici*) and leaf rust (incited by *P. triticina*) resistance while maintaining local adaptation, drought tolerance, and grain quality. However, to achieve these goals require inclusion of diverse novel source of genetic resources, characterization of their genetic diversity and traits, and identification of beneficial genes for their utilization in a wheat breeding program.

It is well known that bringing rich source of genetic resources can be used for enhancing the beneficial genes/variation in modern wheat. However, plant breeders relying on a few varieties led to the loss of genetic diversity in modern wheat (Lage *et al.* 2003; Zhang *et al.* 2005; Bhatta *et al.* 2018c), resulting in a genetic bottleneck. Therefore, wheat breeders have been recovering the lost genetic diversity by utilizing landraces, wild relatives such as *Aegilops* spp., internationally exchanged germplasm, or synthetic hexaploid wheat (Ogbonnaya *et al.* 2013; Dempewolf *et al.* 2017; Bhatta *et al.* 2018c; Morgounov *et al.* 2018). Among these, synthetic hexaploid wheat (2n = 6x = 42, AABBDD: a cross between cultivated *T. turgidum* L.: 2n = 4x =28, AABB, and wild *Aegilops tauschii* Coss.: 2n = 2x = 14, DD) represent a particularly important group of novel genetic resources because of their wide genetic diversity, resistance/tolerance to multiple biotic and abiotic stresses, easy crossing compatibility with wheat, and large number of novel favorable alleles compared to wild relatives (Lage *et al.* 2003; Zhang *et al.* 2005; Zegeye *et al.* 2014; Bhatta *et al.* 2018c, 2018d, 2019a; Morgounov *et al.* 2018).

Unraveling and mining novel genes from rich genetic resources are possible from a genome-wide association study (GWAS). Several studies have used GWAS to unravel novel loci associated with agronomic performance, disease resistance, and quality traits in bread wheat (Neumann *et al.* 2011; Edae *et al.* 2014; Sukumaran *et al.* 2015; Velu *et al.* 2017; Sehgal *et al.* 2017; Lozada *et al.* 2017) and synthetic hexaploid wheat (Ogbonnaya *et al.* 2013; Zegeye *et al.* 2014; Jighly *et al.* 2016; Das *et al.* 2016; Morgounov *et al.* 2018; Bhatta *et al.* 2018a, 2018b, 2018d, 2019a). However, there are many genomic regions/genes for these traits yet to be identified using diverse group of wheat germplasm and new wheat genome sequence information. Therefore, this study was conducted to evaluate phenotypic variation in diverse wheat accessions for agronomic performance, disease resistance, and grain quality; and to identify genomic regions and potential candidate genes from a GWAS. The results from this study provided further insights on genetic characterization of complex traits and assisted in wheat genetic improvement.

## Materials and Methods

### Experimental materials and design

A total of 143 diverse wheat germplasm used in this study represent four major groups. The first group comprised of 13 spring synthetic lines developed by Kyoto University (Japan) from crosses between Langdon durum (*T. turgidum* L.) and 13 diverse accessions of *Ae. tauschii*. The second group comprised of 39 winter synthetics developed by CIMMYT from crosses between five winter durums (Aisberg, Leuc. 84693, Pandur, Ukr-Od. 1530.94, and Ukr-Od. 952.92) and 10 different *Ae. tauschii* accessions. The third group comprised of 14 bread wheat lines originated from USA breeding programs. The fourth group comprised of 77 bread wheat lines developed by KASIB (Kazakhstan and Siberian) breeding program, where14 lines were from Kazakhstan and 63 from Siberia, Russia (Table S1). Genome-wide diversity analysis conducted in these lines showed considerable genetic diversity among lines from different breeding programs, and the population structure analysis identified that these lines can be divided into three subgroups based on the type of wheat and their geographical origin (Bhatta *et al.* 2019b). The idea of selecting diverse germplasm from different breeding programs was to assess their adaptation and performance to a new area (Siberia) as well as to better understand the growing region from already adapted lines in the specific region.

Two years of field experiments (2017 and 2018) were conducted at the research farm located at Omsk State Agriculture University (55°01′122″ N, 73°18′46″ E) under rainfed conditions. The experimental design used in this study was a randomized complete block design with four replications.

### Phenotypic data collection and analysis

Diverse wheat accessions used in this study were evaluated for four diseases [leaf rust, stem rust, powdery mildew (incited by *Blumeria graminis* f. sp. *tritici*), and Septoria tritici blotch (sepf; *Mycosphaerella graminicola*)]; area under disease progress curve (AUDPC) for leaf rust and stem rust; 27 agronomic (grain yield and yield related traits including root traits) and two grain quality (grain protein and gluten content) traits under rainfed conditions. Grain yield related traits were emergence, days to heading, grain length, grain area, grain circularity, grain perimeter, grain weight per plant, grain weight per spike, grains per spike, number of spikes, spike length, spike weight, spike density, and spikelet numbers at maturity, spike harvest index, peduncle length, plant height at maturity, leaf number, number of productive tillers and plants at maturity, harvest index, thousand kernel weight, dry plant weight with roots, root-traits including root diameter, root volume, and total root length. These traits were measured using standard protocol described previously (Bhatta *et al.* 2017, 2018e, 2018b, 2019a; Morgounov *et al.* 2018). Root traits were measured under field conditions 3-4 days after flowering using WinRhizo software (WinRhizo reg. 2009c, Regent Instruments Inc., Quebec City, QC, Canada) as described previously (Bhatta *et al.* 2018b).

An individual analysis of variance for each year was performed due to the significant genotype x year interactions for most of the traits under study (Table 1). Best linear unbiased estimates (BLUEs) were obtained by assuming genotype as a fixed effect and replication as a random effect using PROC MIXED in SAS 9.4 (SAS Institute Inc., Cary, NC). Principal component biplot analysis was performed to understand the association among agronomic, disease resistance, and grain quality traits based on correlation matrix using BLUEs. Variance components were estimated by the restricted maximum likelihood (REML) method assuming a full random model, and entry-mean based broad-sense heritability (*H^2^*) was calculated using following model.

**Table 1:** Basic summary statistics with mean, standard deviation (SD), minimum (Min), and maximum (Max), broad sense heritability (*H^2^*) values, and number of significant marker-trait associations (NMTAs) for 35 traits for 143 diverse synthetic and bread wheat accessions evaluated in 2017 and 2018 at Omsk, Siberia

Traits*^a^*	2017 growing season				2018 growing season				Geno*^b^*	Geno x Year	*H^2^*	NMTAs*^c^*
	Mean	SD	Min	Max	Mean	SD	Min	Max				
Emergence	326	70	61	478	361	61	151	519	***	***	0.64	2
Heading date	41	7	31	57	40	4	31	46	****	***	0.83	9
Powdery mildew	5	2	1	9	4	1	2	7	***	***	0.8	7
Leaf rust severity	25	18	0	70	31	26	0	83	***	***	0.85	12
Leaf rust AUDPC	532	393	0	1609	571	503	0	1696	***	***	0.88	8
Stem rust severity	16	15	0	65	42	23	0	90	***	NS	0.75	5
Stem rust AUDPC	297	304	0	1342	779	524	49.2	2093	***	***	0.79	7
Septoria severity	6	1	3	8	6	1	4	8	*	NS	0.65	3
Grain yield	322	118	53.8	548	390	142	77.2	657	***	***	0.72	5
Leaf number	5	0	4	6	5	0	4	5	*	NS	0.59	4
Grain protein content	17.8	1.56	14.3	22.3	17	1.44	13.9	20.8	***	***	0.89	9
Gluten content	35.6	3.14	28. 7	44.4	33.2	3.81	25.2	43.4	***	***	0.87	7
Grain area	17.4	1.8	13.5	22.5	22.9	1.99	17.5	28.2	***	***	0.82	6
Grain perimeter	17.5	1.25	15.1	21.3	20	1.16	16.9	23	***	***	0.83	6
Grain length	6.81	0.55	5.78	8.58	7.91	0.58	6.44	9.45	***	NS	0.82	11
Grain circularity	0.72	0.03	0.63	0.79	0.72	0.03	0.6	0.78	***	NS	0.37	4
No. of plants	46	13	9	67	51	10	20	76	***	***	0.68	10
No. of spikes	69	22	11	112	78	20	26	128	***	***	0.72	8
Plant weight with roots	5.5	1.76	2.2	11.1	5.46	1.03	2.83	8.42	***	***	0.52	9
No. of tillers	2	0	1	2	2	0	1	2	***	NS	0.33	7
Peduncle length	36.4	6	19.9	48.7	89.8	11. 8	55.2	109	***	***	0.61	8
Plant height	82.7	11.2	44.9	112	39.7	7.27	26.1	54.7	***	***	0.62	5
Spike length	8.4	1.14	6.19	13	9.8	1.18	6.79	12.3	***	***	0.77	10
Spikelet number	14	1	10	18	15	2	11	20	***	***	0.83	12
Spike weight	1.79	0.36	0.96	2.92	2.16	0.38	1.43	3.23	***	NS	0.36	7
Spike density	16.9	2.19	12	21.6	15.9	1.73	11.3	19.8	***	***	0.89	3
Grains per spike	29	6	6	41	35	7	18	53	***	***	0.72	2
Harvest index	0.28	0.07	0.07	0.44	0.27	0.06	0.13	0.41	***	NS	0.57	12
Root length	164	48.1	12.5	311	102	20.9	59.1	161.6	***	***	0.52	7
Grain weight per spike	1.27	0.3	0.25	2.03	1.57	0.35	0.89	2.61	***	NS	0.39	6
Grain weight per plant	1.38	0.41	0.45	2.88	1.48	0.41	0.67	2.83	***	***	0.66	3
Thousand kernel weight	44	4.97	30.7	57.4	44.2	4.34	33.5	54.1	***	***	0.72	10
Spike harvest index	0.71	0.1	0.25	0.83	0.72	0.07	0.47	0.81	*	NS	0.42	8
Root diameter	0.72	0.11	0.49	0.96	0.65	0.06	0.5	0.85	***	**	0.28	3
Root volume	0.72	0.39	0.2	2.04	0.34	0.09	0.16	0.56	***	***	0.61	8

a AUDPC, area under disease progress curve (AUDPC).

b Geno, genotype.

c NMTAs, the total number of significant marker-trait associations identified at the 5% level of significance with a *P* > 1.08 x 10^−6^ (-log_10_*P* = 5.97).

*, **, and *** indicate significant difference at the probability level 0.05, 0.01, and 0.001, respectively. NS, not significant at the probability level 0.05.

$$H^{2}=\frac{\sigma^{2}\text{g}}{\sigma^{2}\text{g}+\frac{\sigma^{2}\text{gxyr}}{\text{y}}+\frac{\sigma^{2}\text{yr}}{\text{yxr}}}$$

where σ^2^_g_, σ^2^_yr_, σ^2^_gxyr_, and σ^2^_e_ are the variance components for genotype, year, genotype × year, and error, respectively, and y and r are the numbers of years and replications, respectively.

### Genotyping and SNP discovery

Genotyping and SNP discovery procedures for all 143 lines were described previously (Bhatta *et al.* 2019b). In brief, genomic DNA was extracted from 2-weeks old leaves using Biosprint 96 Plant Kit (Qiagen) protocol. Genotyping was performed at the Wheat Genetics and Germplasm Improvement Center at Kansas State University, Manhattan, KS using genotyping-by-sequencing (GBS) method. The GBS libraries were constructed in 96-plex following digestion of DNA with two restriction enzymes (*PstI* and *MsPI*), and pooled libraries were sequenced using Illumina HiSeq (Illumina, Inc., San Diego). SNP discovery was performed using TASSEL v.5.2.40 GBS v2 Pipeline (Glaubitz *et al.* 2014) with a physical alignment to the Chinese spring genome sequence (RefSeq v1.0) (International Wheat Genome Sequencing Consortium 2018). The identified SNPs with minor allele frequency (MAF) of more than 5% and missing data frequency of less than 20% were retained for the analysis (Bhatta *et al.* 2018c, 2019b).

### Genome-wide association study

The multi-locus mixed linear model using FarmCPU (Fixed and random model Circulating Probability Unification) algorithm implemented in R package (Liu *et al.* 2016a) was used for the association mapping. Population structure analysis performed in our recent study identified that the 143 lines can be divided into three sub-population (*Q*_1-3_) using Bayesian clustering algorithm (Bhatta *et al.* 2019b). Therefore, the first three *Q*_1-3_ matrices were used for controlling the population structure in GWAS analysis. In the FarmCPU model, *Q* matrix was used as a fixed effect whereas kinship matrix (K, controlling genetic relatedness) was used as a random effect (Liu *et al.* 2016a). BLUEs calculated for each trait in each year were taken as phenotype and 46,268 GBS-derived SNPs (Table S2) were taken as a genotype for the GWAS. The model fit was tested using the quantile-quantile plot considering the deviation of the observed test statistics values from the expected test statistics values (Figure S1). The identified MTAs were corrected for multiple testing and the MTAs were tested against a Bonferroni correction at the 5% level of significance with a *P* > 1.08 × 10^−6^ (-log_10_*P* = 5.97). The percent of phenotypic variance explained by each significant SNP was estimated using multiple-linear mixed model as described previously (Bhatta *et al.* 2018d).

### Linkage disequilibrium estimation

Linkage disequilibrium (LD) among markers was estimated using the full matrix and sliding window size of 50 in Tassel V5.2.52 (Bradbury *et al.* 2007), using 46,268 SNPs, for the A, B, D, and entire genomes. LD was calculated as the squared allele frequency correlations (*r*^2^). Pairwise LD *r^2^* values were plotted against the corresponding physical distance, and a non-linear regression model was fitted to estimate the genome-wide LD decay (Hill and Weir 1988; Remington *et al.* 2001). The critical value of *r^2^* beyond which the LD was likely to be caused by linkage was set at *r^2^* = 0.2. The average LD decay of the association mapping panel was determined as the point at which LD curve intercepts the critical *r^2^*.

### Putative candidate gene annotation

The putative genes underlying the significant SNPs and their annotations were obtained from the IWGSC RefSeq V1.0 annotations provided for the Chinese spring wheat (International Wheat Genome Sequencing Consortium 2018). The predicted effect of the SNPs on the protein function was obtained from Variant Effect Predictor by EnsemblPlants for wheat (http://plants.ensembl.org/Triticum_aestivum/Tools/VEP/).

### Data availability

Supplementary files including genotyping-by-sequencing derived SNP marker data are available in figshare at https://gsajournals.figshare.com/s/6434bc736b63a242e546. Figure S1 contains Manhattan and quantile-quantile (Q-Q) plots for 35 traits in 143 diverse wheat accessions obtained from a genome-wide association study. Figure S2 contains principal component biplot analysis among agronomic, disease resistance, and grain quality traits in 143 diverse wheat accessions. Figure S3 contains the physical distribution of 46,268 genotyping-by-sequencing derived SNPs within a 1-Mb window size on 21 chromosomes (Chr) of 143 diverse accession of wheat. Table S1 contains details of the 143 diverse wheat accessions used in this study. Table S2 contains genotype-by-sequencing derived SNPs identified in 143 diverse wheat lines. Table S3 contains details of significant markers associated with 35 traits in 143 diverse wheat accessions grown in two seasons (2017 and 2018) in Siberia. Table S4 contains details of potential candidate gene functions harboring SNPs affecting agronomic, disease resistance, and grain quality traits from two years (2017 and 2018) of experiments conducted in Siberia using 143 diverse wheat accessions. Table S5. Genomic fingerprinting of 143 diverse accessions of wheat showing the distribution of favorable (1), unfavorable (0) and heterozygote (0.5) alleles of significant marker-trait associations identified in this study. The synthetic and bread wheat seeds are available upon request. Supplemental material available at figshare: https://doi.org/10.25387/g3.9943682.

## Results

### Phenotypic evaluation

The combined analysis of variance (ANOVA) revealed significant cross-over genotype x year interaction for most of the traits under study (Table 1). Therefore, individual ANOVA was performed and it revealed significant genetic variation for all traits (Table 1). Variation for grain yield ranged from 54 to 549 g m^-2^ with an average of 322 g m^-2^ in 2017 and it ranged from 77 to 657 g m^-2^ with an average of 391 g m^-2^ in 2018. Similarly, variation for grain protein content ranged from 14 to 22% with an average of 18% in 2017 and it ranged from 14 to 21% with an average of 17% in 2018 (Table 1).

This study identified several lines with low disease severity scores.. For instance, seven, 10, and three lines had very low disease severity scores against leaf rust (2017ENTRY # 66: RBOT, 69: ‘Rollag’, 71: Sadin,75: Kelby, 77: Brennan, 82: Advance, and 155: ‘Novosibirskaya 16’), stem rust (2017ENTRY # 12: ‘AISBERG/AE.SQUARROSA_511’, 63: ‘LANGDON/KU-2092’, 69, 72: ‘Tom’, 75, 77, 79: ‘SY TYRA’, 82, 155, and 156: ‘Novosibirskaya 41’), and powdery mildew (2017ENTRY # 69, 74: ‘Knudson’, and 114: ‘OmGAU-90’), respectively, in 2017 (Table S1). One line (2017ENTRY # 69) had the lowest severity scores against all these diseases, and five lines (2017ENTRY # 69, 75, 77, 82, and 155) had lower disease severity scores against both leaf and stem rusts. In 2018, we had 14 lines (2017ENTRY # 59, 75, 76, 77, 79, 87, 91, 102, 105, 106, 111, 112, 152, and 153) with the lowest disease severity scores against leaf rust and one line (2017ENTRY #24: ‘AISBERG/AE.SQUARROSA_511’) had the lowest severity score against stem rust. Based on two years of data, we have recommended top five ranking lines (2017ENTRY # 91 ‘Lutestsens 7/04-4’, 94: ‘Element 22’, 112: ‘Lutestsens 15-14’, 158: ‘Lut. 3/04-21-11’, and 164: ‘Silach’) that had grain yield > 453 g/m^2^, protein content >15.5%, thousand kernel weight > 41.5 g, spike harvest index >0.74, and low to moderate level of disease severity scores against multiple foliar fungal diseases (Table S1).

Broad sense heritability of the traits ranged from low to high heritability (Table 1). The highest heritability was observed for grain protein content (*H^2^* = 0.89) and spike density (*H^2^* = 0.89), followed by leaf rust AUDPC (*H^2^* = 0.88), gluten content (*H^2^* = 0.87), leaf rust severity score (*H^2^* = 0.85), and heading date (*H^2^* = 0.83) whereas the lowest heritability was observed for root diameter (*H^2^* = 0.28). The heritability observed for grain yield (*H^2^* = 0.75) and thousand kernel weight (*H^2^* = 0.72) in this study were also in the high heritability range (*H^2^* > 0.70).

A principal component analysis (PCA) based on correlation matrix was performed to investigate the relationship among all 35 traits (Figure S2). Approximately, 68.4% of the total variation in 143 lines were explained by the first four PCs. The first two PCs were predominantly associated with agronomic and grain quality traits, and it contributed ∼54% of the total variation in the data. Wheat rust diseases such as leaf and stem rusts represented the third PC, and it contributed 8.9% of the total variation. Similarly, root traits such as total root length and root volume were associated with the fourth PC, and it contributed 5.5% of the total variation.

### Marker density and linkage disequilibrium

A total of 192,876 GBS derived SNPs were obtained based on the Chinese Spring RefSeq v1.0. After removing SNPs with MAF <5% and missing data >20%, 46,268 SNPs were retained for subsequent analysis (Table S2). The marker density for the A, B, and D genome were 20.2, 20.8, and 20.2 Mb per SNPs, respectively (Figure S3).

**Figure 1:**
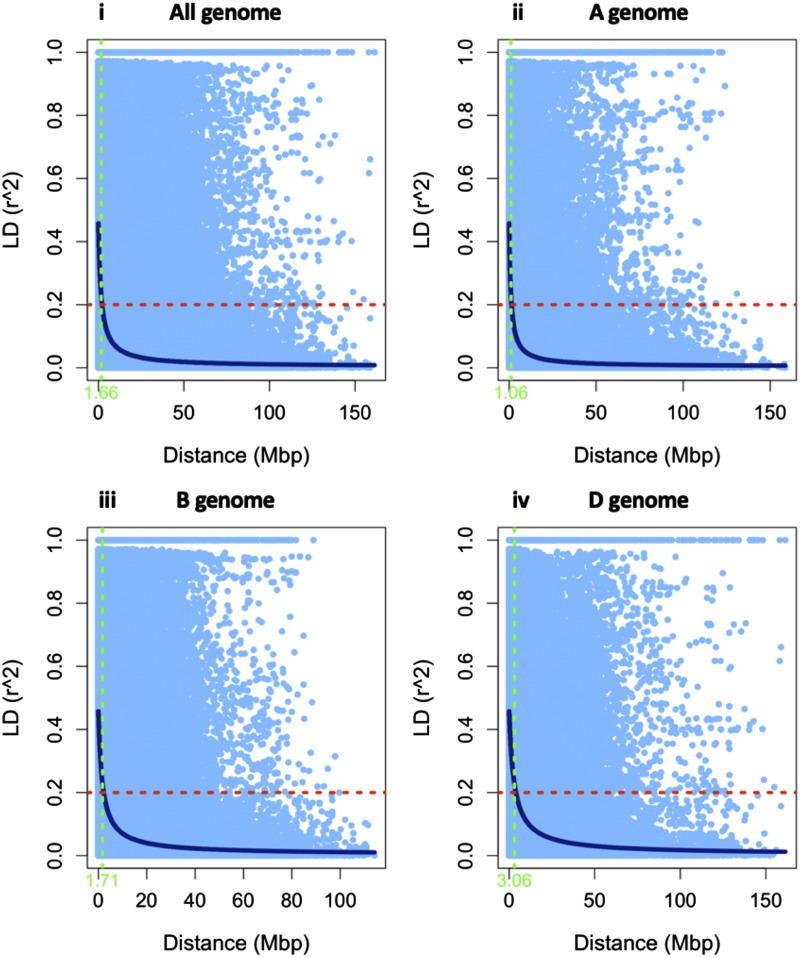
Scatter plot showing the linkage disequilibrium (LD) decay across the chromosomes for 143 diverse wheat accessions. The genetic distance in mega base pair (Mbp) is plotted against the LD estimate (*r^2^*) for pairs of SNPs. The dotted red line shows the threshold LD value at *r^2^* = 0.2 and dotted green line shows the average LD decay point at which LD curve intercepts the critical *r^2^*. (**i**) Genome-wide average LD decay plot using all genomes; (**ii**) LD decay plot of the A genome; (**iii**) LD decay plot of the B genome; and (**iv**) LD decay plot of the D genome.

A total of 46,268 markers were used to evaluate LD decay for the A, B, D, and the whole genome. The scatter plot of *r^2^* against physical distance showed that the LD decay with increasing physical distance. The average LD decayed below *r^2^* = 0.2 for the entire genome was 1.06 Mb where, the greatest extent of LD was observed on the D genome (3.06 Mb), followed by the B (1.71 Mb) and A (1.06 Mb) genomes (Figure 1).

### Genome-wide association study

**Figure 2:**
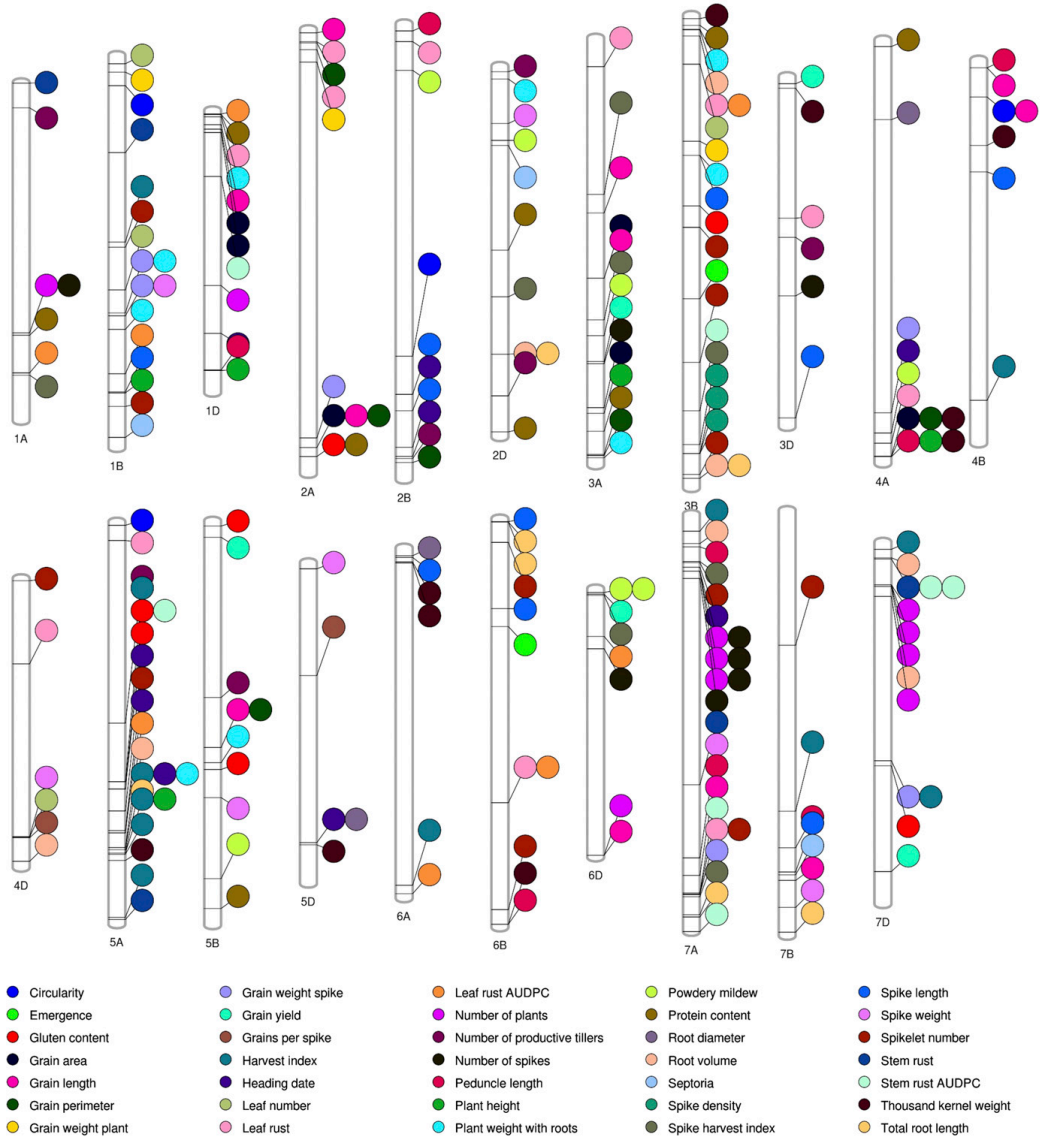
Significant marker-trait associations (*P* > 1.08 × 10^−6^) identified on each chromosome for agronomic, disease resistance, and grain quality traits obtained from the genome-wide association study of 143 synthetic and bread wheat lines grown in 2017 and 2018 growing seasons in Siberia.

A GWAS identified a total of 243 significant SNPs (*P* > 1.08 × 10^−6^) that were associated with 35 traits including agronomic, disease resistance, and grain quality traits, (Figure 2, Tables 1 and S3). These significant SNPs were distributed across all 21 chromosomes of wheat, and the phenotypic variance explained (PVE) by these SNPs ranged from 0.3 to 25.0%. The highest number of MTAs were identified on the B genome (85 MTAs) followed by the A (72 MTAs), and the D (62 MTAs) genomes of wheat. Results of specific group of traits will be discussed hereafter.

### Agronomic performance traits

A total of 185 MTAs were identified for the agronomic performance traits evaluated in this study (Figure 2). The MTAs were distributed across all chromosomes with PVE ranging from 0.3 to 25% (Table S3). Of the 185 MTAs, 72, 68, and 45 MTAs were detected on the A, B, and D genome, respectively. The highest number of MTAs were observed for harvest index (12) and spikelet number (12), followed by grain length (11), thousand kernel weight (10), spike length (10), and number of plants (10), while the lowest number of MTAs were observed for emergence (2) and grains per spike (2).

Agronomic performance traits were divided into five sub-groups which are yield component, grain related, spike productivity, phenological, and root traits. For yield component traits, a total of 27 MTAs were identified on chromosomes 1B, 3A, 3B, 3D, 4A, 4B, 5A, 5B, 5D, 6A, 6B, 6D, 7A, 7B, and 7D (Figure 2) with PVE ranging from 2.5 to 16.5% (Table S3). Of these, five, 10, and 12 MTAs were associated with grain yield, thousand kernel weight, and harvest index, respectively. Similarly, for grain related traits, a total of 38 MTAs were identified on chromosomes 1B, 1D, 2A, 2B, 3A, 3B, 4A, 4B, 4D, 5A, 5B, 5D, 6D, 7A and 7B with PVE ranging from 0.3 to 19.6%. Of these, 11, six, six, six, two, three, and four MTAs were associated with grain length, grain perimeter, grain area, grain weight per spike, grains per spike, grain weight per plant, and grain circularity. For spike productivity traits, a total of 48 MTAs were identified on chromosomes 1A, 1B, 2B, 2D, 3A, 3B, 3D, 4B, 4D, 5A, 5B, 5D, 6A, 6B, 6D, 7A, and 7B with PVE ranging from 2.3 to 21.0%. Of these, eight, 12, 10, seven, eight, and three MTAs were associated with number of spikes, spikelet numbers, spike length, spike weight, spike harvest index, and spike density, respectively. For phenological traits, a total of 54 MTAs were identified on chromosomes 1B, 1D, 2B, 3A, 4A, 5A, 5D, and 7A (Figure 2) with PVE ranging from 1 to 13% (Table S3). Of these, two, nine, four, 10, seven, eight, five, and nine MTAs were associated with emergence, heading date, number of plants, number of productive tillers, peduncle length, plant height, and dry plant weight with roots, respectively (Table S3). A total of 18 MTAs were identified for root traits on chromosomes 2D, 3B, 4A, 4D, 5A, 5D, 6A, 6B, 7A, 7B, and 7D (Figure 2) with PVE ranging from 0.3 to 25.0% (Table S3). Of these, seven, eight, and three MTAs were associated with total root length, root volume, and root diameter, respectively.

### Disease resistance traits

A total of 42 MTAs were identified for six disease-related traits distributed on all chromosomes except for chromosomes 4B and 5D (Figure 2) with PVE ranging from 1.0 to 14.0% (Table S3). In detail, 12, five, seven, three, eight, and seven MTAs were associated with leaf rust, stem rust, powdery mildew, Septoria, leaf rust AUDPC, and stem rust AUDPC scores, respectively.

### Grain quality traits

Grain protein concentration and gluten content are two grain quality traits measured in this study. A total of 16 MTAs were identified for grain quality-related traits on chromosomes 1A, 1D, 2A, 2D, 3A, 3B, 4A, 5A, 5B, and 7D (Figure 2) with PVE ranging from 1.9 to 13.9% (Table S3). Of these, nine and seven MTAs were associated with grain protein and gluten content, respectively.

### Putative candidate gene annotation

The functional annotation of genes underlying significant SNPs was identified through the IWGSC RefSeq v1.1 annotation and the impact of the genes was identified through variant effect predictor from EnsemblPlants (http://plants.ensembl.org/Tools/VEP). A total of 116 MTAs were found within genes and of these, 21 MTAs were annotated as a missense variant and had a moderate impact (Table S4). Moderate impact genes were found for several traits such as grain length, grain perimeter, heading date, leaf rust AUDPC, number of productive tillers, number of spikes, peduncle length, plant weight with roots, protein content, Septoria, spike length, spike weight, spikelet number, and thousand kernel weight. The detailed annotations of these gene-IDs are provided in Table 2.

**Table 2:** List of important genes underlying significant marker-trait associations for several traits under study whose annotation showed moderate impact and consequence as missense variant

Trait	SNP-ID	Gene-ID	Annotation
Grain length	S3A_495499647	TraesCS3A01G269400.1	Transmembrane protein, putative
Grain perimeter	S2A_24003058	TraesCS2A01G056500.1	Sulfotransferase
Heading date	S5A_549160106	TraesCS5A01G344900.1	SAUR-like auxin-responsive protein family
Leaf rust	S1D_6789756	TraesCS1D01G013500.1	12-oxophytodienoate reductase-like protein
Leaf rust	S3A_50747630	TraesCS3A01G078500.1	E3 Ubiquitin ligase family protein
Leaf rust AUDPC	S1B_560253737	TraesCS1B01G333800.1	GDSL esterase/lipase
Number of productive tillers	S2D_10070953	TraesCS2D01G021900.1	NBS-LRR-like resistance protein
Number of productive tillers	S3D_282956863	TraesCS3D01G211600.1	Protein CHUP1, chloroplastic
Number of spikes	S6D_104966449	TraesCS6D01G136500.1	Methyltransferase-like protein
Penduncle length	S1D_456199885	TraesCS1D01G381100.1	Lipid transfer protein
Plant weight with roots	S5B_426587161	TraesCS5B01G245400.1	Protein DETOXIFICATION
Protein content	S2D_643258624	TraesCS2D01G582500.1	Kinase, putative
Protein content	S2D_323648225	TraesCS2D01G264700.1	NBS-LRR disease resistance protein
Septoria	S7B_643501119	TraesCS7B01G379200.1	F-box/RNI-like/FBD-like domains-containing protein
Spike harvest index	S2D_405601286	TraesCS2D01G316300.1	Kinesin-like protein
Spike length	S6B_4874543	TraesCS6B01G007400.1	Amino acid transporter
Spike weight	S5B_487919184	TraesCS5B01G303600.1	Sister chromatid cohesion 1 protein 3
Spikelet number	S3B_807353230	TraesCS3B01G577400.1	Protein TolB
Spikelet number	S3B_372895173	TraesCS3B01G237700.1	Zinc finger protein
Thousand kernel weight	S6A_26974912	TraesCS6A01G052100.2	Kinase family protein
Thousand kernel weight	S5D_498302498	TraesCS5D01G449100.1	RNA-binding protein 47A

### Multi-trait and stable marker trait associations

Multi-trait MTAs are the common genomic regions controlling multiple traits. The present study identified 23 multi-trait (group affects 2-4 traits) MTAs located on chromosomes 1A, 1B, 2A, 2D, 3B, 4A, 4B, 5A, 5B, 5D, 6B, 7A, and 7D (Table 3) and of these 23 MTAs, 18 were within genes. For example, a SNP at S1A_440183259 on chromosome 1A was significantly associated with number of plants and spikes and was present in rhomboid family protein (TraesCS1A01G248200.1) gene. Also, a SNP at S2A_734585818 bp on 2A chromosome was significantly associated with grain area, length, and perimeter and was present in 70 kD heat shock protein (TraesCS2A01G506900.1) gene. Stable markers (marker consistently identified in both years) were identified for thousand kernel weight (S4A_732725628) on chromosome 4A at 733 Mb, powdery mildew (S6D_5824400) on chromosome 6D at 582 Mb, and stem rust AUDPC (S7D_29550951) on chromosome 7D at 296 Mb (Table S3).

**Table 3:** Multi-trait marker-trait associations with phenotypic variance explained (PVE) and SNP effect identified from genome-wide association study on 143 diverse accessions of wheat

Multiple traits	SNP	PVE	SNP effect	Gene-ID	Gene annotation
Number of plants and spikes	S1A_440183259	4.56-7.12	−3.4 to -6.96	TraesCS1A01G248200.1	Rhomboid family protein
Grain weight per spike and plant weight with roots	S1B_453188672	4.16-5.78	0.06 to 0.25	TraesCS1B01G257400.1	pale cress protein (PAC)
Grain weight per spike and spike weight	S1B_457535596	5.27-6.78	0.07 to 0.15	TraesCS1B01G260200.1	Beta-1,3-galactosyltransferase-like protein
Grain length, perimeter, and area	S2A_734585818	6.36-14.03	0.37 to 1.12	TraesCS2A01G506900.1	70 kD heat shock protein
Protein and gluten content	S2A_750550946	5.35-7.72	0.55 to 1.21	TraesCS2A01G536200.1	Sulfotransferase
Protein and gluten content	S2A_750550946	—	—	TraesCS2A01G536300.1	Serine/threonine-protein kinase
Total root length and root volume	S2D_506778844	9.22-16.79	−0.24 to -31.41	TraesCS2D01G395500.1	Ankyrin repeat protein-like
Leaf rust and leaf rust AUDPC	S3B_36628392	1.72-2.16	−7.58 to -137.81	—	—
Total root length and root volume	S3B_814148012	8.87-10.14	0.15 to 19.81	TraesCS3B01G587900.1	Disease resistance protein RPM1
Thousand kernel weight, grain area and grain perimeter	S4A_732725628	9.93-11.46	−0.46 to -2.21	—	—
Plant height, peduncle length, and thousand kernel weight	S4A_732825683	8.81-10.83	−2.22 to -4.74	—	—
Grain length and grain circularity	S4B_64816370	8.2-11.46	0.01 to -0.23	—	—
Gluten content and stem rust AUDPC	S5A_471711779	5.86-6.98	−1.44 to 166.41	TraesCS5A01G256100.1	Zinc transporter, putative
Total root length, heading date, plant weight with roots, and harvest index	S5A_584618691	8.99-12.3	0.04 to -12.9	TraesCS5A01G387500.1	Cytosolic Fe-S cluster assembly factor NAR1
Harvest index and plant height	S5A_584712144	6.35-12.58	−0.03 to 5.02	TraesCS5A01G387800.1	RNA binding family protein isoform 1
Harvest index and plant height	S5A_584712144	—	—	TraesCS5A01G387900.1	Glycosyltransferase
Grain length and grain perimeter	S5B_399398211	8.94-6.92	0.18 to 0.5	—	—
Heading date and root diameter	S5D_494053024	4.33-11.15	−1.1 1to -0.05	TraesCS5D01G441300.1	Lipid transfer protein
Leaf rust and leaf rust AUDPC	S6B_501107290	6.1-8.21	7.46 to 125.57	TraesCS6B01G276900.1	Phosphatidic acid phosphatase
Leaf rust and leaf rust AUDPC	S6B_501107290	—	—	TraesCS6B01G277000.1	Vesicle-associated 1-1-like protein
Number of plants and spikes	S7A_100624533	9.99-12.33	8.59 to 12.94	TraesCS7A01G147500.1	Basic helix-loop-helix transcription factor
Number of plants and spikes	S7A_100624536	9.99-12.33	8.59 to 12.94	—	—
Number of plants and spikes	S7A_100624582	9.99-12.33	8.59 to 12.94	—	—
Spikelet number and leaf rust	S7A_670904777	4.4-4.68	0.38 to 10.24	—	—
Stem rust and stem rust AUDPC	S7D_29550951	0.95-9.75	−4.82 to -191.09	TraesCS7D01G055000.1	Acid invertase 1
Harvest index and grain weight per spike	S7D_385718003	4.15-9.27	0.01 to 0.07	TraesCS7D01G304100.1	Polyribonucleotide nucleotidyltransferase

### Genomic fingerprinting and favorable alleles distribution in synthetic and bread wheat accessions

**Figure 3:**
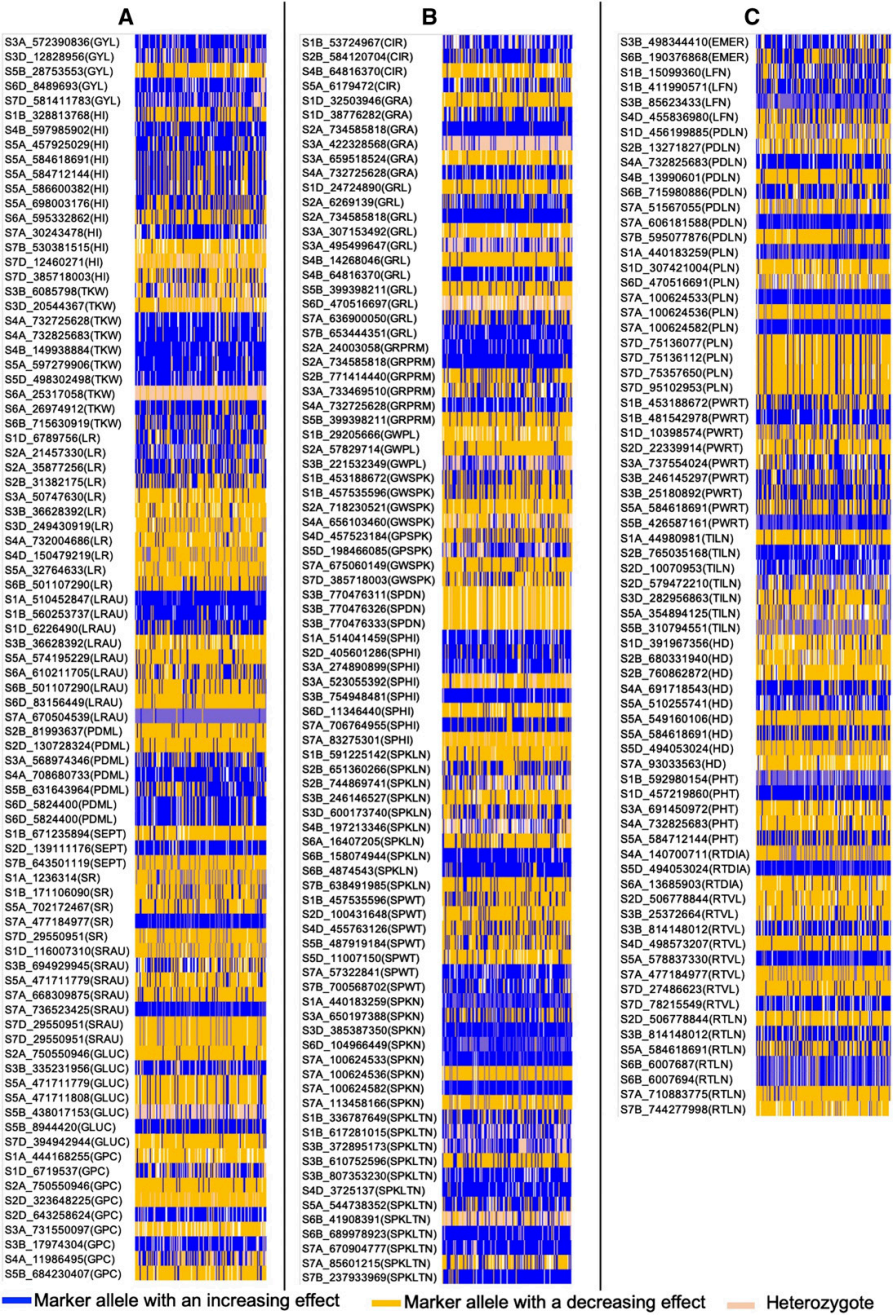
Genomic fingerprinting of 143 diverse accessions of wheat lines (x-axis) showing distribution of favorable (1), unfavorable (0) and heterozygote (0.5) alleles for markers that were significantly associated with grain yield components (A), disease resistance (A), quality traits (A), grain and spike related traits (B), phenological and root traits (C). Traits are grain circularity (CIR), emergence (EMER), gluten content (GLUC), grain area (GRA), grain length (GRL), grain perimeter (GRPRM), grain weight per plant (GWPL), grain weight per spike (GWSPK), grain yield (GYL), grains per spike (GPSPK), harvest index (HI), heading date (HD), leaf number (LFN), leaf rust (LR), leaf rust AUDPC (LRAU), number of plants (PLN), number of productive tillers (TILN), number of spikes (SPKN), peduncle length (PDLN), plant height (PHT), plant weight with roots (PWRT), powdery mildew (PDML), grain protein content (GPC), root diameter (RTDIA), root volume (RTVL), Septoria (SEPT), spike density (SPDN), spike harvest index (SPHI), spike length (SPKLN), spike weight (SPWT), spikelet number (SPKLTN), stem rust (SR), stem rust AUDPC (SRAU), thousand kernel weight (TKW), and total root length (RTLN).

Genomic fingerprinting for 243 MTAs with the distribution of favorable, unfavorable, and heterozygote alleles in 143 diverse wheat lines is shown in Figure 3 and Table S5. Favorable alleles in this study were defined as the alleles of MTAs that had an increasing effects on agronomic performance, disease resistance, and grain quality traits in wheat accessions. For grain yield and yield components, 18 out of 27 fingerprinted MTAs had favorable alleles in more than 55% of lines (Figure 3A). Similarly, 15/38, 24/48, 20/54, 5/18, 13/42, and 5/16 fingerprinted MTAs, respectively, for grain related-, spike productivity-, phenological-, root-, disease resistance-, and grain quality-traits, had favorable alleles in more than 50% of lines (Figure 3 and Table S5). Top five lines (2017ENTRY # 91, 94, 112, 158, and 164) recommended in this study had 92 to 102 MTAs with favorable alleles.

**Figure 4:**
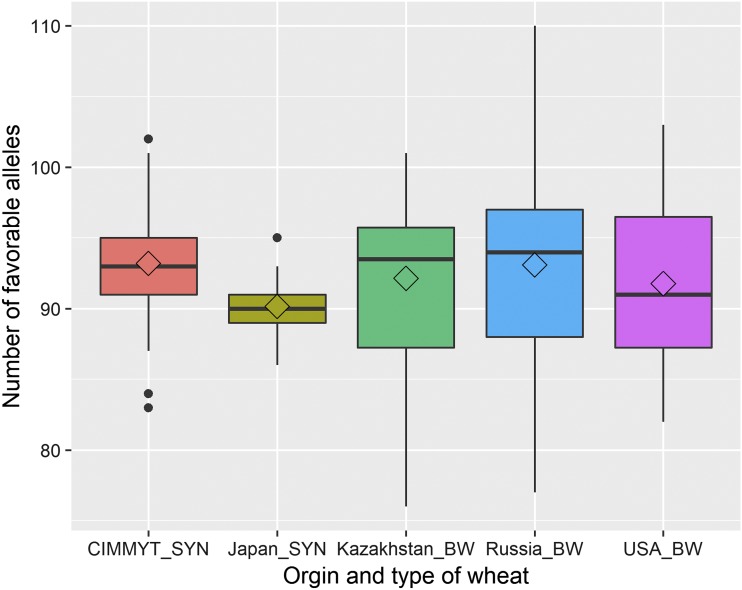
Distribution of favorable alleles in 143 synthetic (SYN) and bread wheat (BW) accessions derived from five different origins.

A wide range of distribution of favorable alleles between synthetic and bread wheat accessions was observed (Figure 4 and Table S5). For example, synthetic wheat originated from CIMMYT and Japan had favorable alleles ranging from 83 to 102 and 86 to 95, respectively. Bread wheat originated from Kazakhstan, Russia, and USA had favorable alleles ranging from 76 to 110. This study identified a total of 62 MTAs on the D genome where 42 of them are novel MTAs. Of these 42 novel MTAs, synthetic wheat originated from CIMMYT and Japan had favorable alleles ranging from 12 to 26 and 20 to 28, respectively (Table S5).

## Discussion

Multiple traits such as grain yield and disease related traits are complex and quantitative in nature, and are largely governed by multiple genes and environmental factors . Genetic dissection of these traits is still a challenge, however, the recent advances in sequencing technologies has provided better resolution in deciphering these traits. Furthermore, the utilization of recently published fully annotated wheat reference genome will enhance wheat genetic improvement. The current study used diverse accessions of bread and synthetic wheat to understand the genetic architecture of multiple traits that are collected in most of the breeding programs.

### Phenotypic evaluation

In this study, diverse wheat accessions showed significant level of variation for agronomic, disease, and quality traits with low to high heritability. Significant cross-over genotype by year interaction was observed for most of the traits, indicating high influence of environmental variation and trait instability/complexity (meaning the genes are greatly affected by the environment). Previous studies have also identified these traits as complex traits with similar heritability (Ogbonnaya *et al.* 2013; Bhatta *et al.* 2017, 2018b; Sehgal *et al.* 2017). The grain yield heritability ranged from 0.20 to 0.75 (Bhatta *et al.* 2018b; Sun *et al.* 2019) in previous studies was also in line with our findings. We have recommended five top performing lines with high yield, multiple disease resistance, and better grain quality for its direct use in a breeding program.

### Linkage disequilibrium

A recent study on a diverse panel of 166 wheat lines from China have identified the LD decay at 6 Mb, 4 Mb, 11 Mb, and 8Mb for the A, B, D, and the entire genomes, respectively (Liu *et al.* 2017b). The LD decay identified in our study was lower than this recent study in wheat (Liu *et al.* 2017b). However, the highest LD decay on the D genome observed in our study was consistent with many previous reports (Edae *et al.* 2014; Sukumaran *et al.* 2015; Liu *et al.* 2017b), indicating that fewer markers are needed for GWAS on the D genome compared to either A or B genome. Relatively higher LD decay on the D genome may be attributed to the inclusion of diverse accessions of *Ae. tauschii* primarily from synthetic hexaploid wheat.

### Genome-wide association study

Genome-wide association study was performed on the 35 traits with the use of ∼46,000 GBS-derived SNPs based on the multi-locus mixed linear model implemented in FarmCPU algorithm that remove the confounding between testing markers and kinship that results in false negatives, prevents model overfitting, and control false positives simultaneously (Liu *et al.* 2016a). The present study identified many significant SNPs (on all chromosomes) and related putative candidate genes associated with agronomic, disease resistance, and grain quality traits by employing the GWAS approach.

### MTAs for agronomic performance traits

Previous GWAS and QTL (quantitative trait loci) mapping studies found QTL/MTAs for grain yield on chromosomes 3A (Neumann *et al.* 2011; Bordes *et al.* 2014; Hoffstetter *et al.* 2016; Sehgal *et al.* 2017; Bhatta *et al.* 2018b), 3D (Bordes *et al.* 2014; Bhatta *et al.* 2018b), 5B (Pinto *et al.* 2010; Neumann *et al.* 2011; Bordes *et al.* 2014; Edae *et al.* 2014; Sukumaran *et al.* 2015), and 6D (Bhusal *et al.* 2017). However, it is difficult to align our findings with previous studies due to the absence of precise physical location information in published studies, use of different marker systems (90K SNP, short sequence repeat, and diversity arrays technology marker), and different versions of wheat reference genome used than the recent IWGSC RefSeq v1.0 (Liu *et al.* 2017b; Bhatta *et al.* 2018b). Despite this fact, the identification of MTAs on the same chromosome as earlier studies could provide confidence on these associations. Since, we cannot directly compare with QTL reported by other previous studies, we have assumed the significant MTAs identified in this study as the novel marker if it was not reported on the same chromosome in previous studies. For example, to the best of our knowledge, an MTA identified for grain yield on chromosome 7D in this study has not been previously reported and it is potentially a novel MTA responsible for controlling grain yield in wheat.

Similarly, we identified several QTL for thousand kernel weight, which were reported earlier on chromosomes 3B (Golabadi *et al.* 2011; Sukumaran *et al.* 2018; Bhatta *et al.* 2018b), 3D (Liu *et al.* 2018), 4A (Bhatta *et al.* 2018b), 4B (Pinto *et al.* 2010; Bhatta *et al.* 2018b), 5A (Sukumaran *et al.* 2015; Guan *et al.* 2018), 5D (Liu *et al.* 2018), 6A (Sukumaran *et al.* 2015; Guan *et al.* 2018), and 6B (Assanga *et al.* 2017), suggesting the important genomic regions governing thousand kernel weight. For harvest index, we identified three novel MTAs for increasing harvest index on chromosomes 4B and 7D. Other MTAs identified for harvest index were reported earlier on chromosomes 1B (Acuña-Galindo *et al.* 2015; Sukumaran *et al.* 2015), 5A (Acuña-Galindo *et al.* 2015), 6A (Golabadi *et al.* 2011; Assanga *et al.* 2017), 7A (Acuña-Galindo *et al.* 2015), and 7B (Neumann *et al.* 2011; Golabadi *et al.* 2011; Bhatta *et al.* 2018b).

Understanding the genetic architecture of grain is very important for determining the grain yield of any crop. However, there is limited study on understanding genetic architecture and complexity of grain related traits (Arora *et al.* 2017). This study evaluated several grain related traits and identified several associated MTAs for grain related traits such as grain length, perimeter, area, grains per spike, grain weight per spike, etc. Specifically, this study identified 17 novel MTAs associated with improving architecture traits in wheat on chromosomes 3A and 6D for grain length, on chromosomes 3A and 4A for grain perimeter, on chromosomes 1D and 3A for grain area, on chromosome 7D for grain weight per spike, on chromosomes 1B, 2A, and 3B for grain weight per plant, and on chromosomes 1B, 2B, 4B, and 5A for grain circularity. Remaining MTAs identified for grain related traits were previously reported on chromosomes 1D (Xiao *et al.* 2011), 2A (Xiao *et al.* 2011), 4B (Breseghello and Sorrells 2007; Sun *et al.* 2008b; Xiao *et al.* 2011) 5B (Dholakia *et al.* 2003; Breseghello and Sorrells 2007; Xiao *et al.* 2011), 7A (Xiao *et al.* 2011), and 7B (Dholakia *et al.* 2003) for grain length; on chromosomes 2A (Xiao *et al.* 2011), 2B (Campbell *et al.* 1999), and 5B (Xiao *et al.* 2011) for grain perimeter; on chromosomes 2A (Campbell *et al.* 1999) and 4A (Xiao *et al.* 2011) for grain area; on chromosomes 1B (Bhusal *et al.* 2017), 2A (Bhusal *et al.* 2017), 4A (Guan *et al.* 2018), and 7A (Guan *et al.* 2018) for grain weight per spike; on chromosomes 4D (Zhang *et al.* 2010) and 5D (Liu *et al.* 2006; Zhang *et al.* 2010) for grains per spike. This study provided better understanding of genetic architecture of grain related traits and the results of these traits will be useful in improving grain yield in wheat and related cereal crops.

Spike-related traits play an important role in determining the yield potential in wheat (Reynolds *et al.* 2009). This study evaluated six spike-related traits to unravel the genetic basis of spike architecture in wheat. We identified 28 novel MTAs for spike-related traits such as number of spikes (3), spikelet numbers (6), spike density (3), spike length (7), spike weight (4), and spike harvest index (5). Remaining identified MTAs for spike productivity traits were previously reported on chromosomes 3D (Liu *et al.* 2006) and 7A (Guan *et al.* 2018) for number of spikes; on chromosomes 4D (Chu *et al.* 2008), 5A (Li *et al.* 2002), and 7A (Li *et al.* 2002) for spikelet numbers; on chromosomes 1B (Li *et al.* 2002) 3D (Chu *et al.* 2008), and 4B (Liu *et al.* 2006) for spike length; on chromosomes 1B (Golabadi *et al.* 2011; Acuña-Galindo *et al.* 2015), 5B (Acuña-Galindo *et al.* 2015), and 7B (Golabadi *et al.* 2011) for spike weight; on chromosomes 3B (Golabadi *et al.* 2011) and 7A (Golabadi *et al.* 2011) for spike harvest index.

Phenological traits such as emergence, number of leaves per plants, number of plants/m^2^, tillers per plant, peduncle length, and dry plant weight have rarely been studied for QTL study. All the MTAs (40) identified for these traits have not been previously reported and they are potentially novel MTAs controlling these traits. For other phenological traits such as plant height and heading date, most of the QTL identified in this study have been reported previously on chromosomes 1B, 3A, and 5A (Chu *et al.* 2008; Sukumaran *et al.* 2015; Guan *et al.* 2018). However, seven MTAs identified for plant height (chromosomes 1D and 4A) and heading date (chromosomes 1D, 2B, 4A, 5D, and 7A) have not been previously reported, and are potentially novel MTAs governing these traits.

Root traits are complex, labor intensive, and expensive traits to measure. Limited studies have been conducted on the field based root traits analysis in wheat (Bhatta *et al.* 2018b) and a few QTL have been identified (Canè *et al.* 2014; Maccaferri *et al.* 2016; Bhatta *et al.* 2018b). The current study examined the roots traits such as root length, root volume, and root diameter after 3-4 days of flowering. This study identified nine novel MTAs associated with root traits on chromosomes 2D, 3B, 4A, 4D, 5D, 6A, and 7D. However, several MTAs identified in our study have been previously reported for root length on chromosomes 3B (Maccaferri *et al.* 2016; Bhatta *et al.* 2018b), 5A (Maccaferri *et al.* 2016), 6B (Maccaferri *et al.* 2016), 7A (Maccaferri *et al.* 2016; Bhatta *et al.* 2018b), and 7B (Maccaferri *et al.* 2016), and for root volume on chromosomes 5A and 7A (Maccaferri *et al.* 2016).

### MTAs for disease resistance traits

Genetic resistance is one of the sustainable approaches for controlling disease. This study have identified several MTAs for leaf rust, stem rust, powdery mildew, and Septoria similar to previous studies, where they have identified QTL/genes for rusts on all 21 chromosomes (Rosewarne *et al.* 2012; McIntosh *et al.* 2014; Kertho *et al.* 2015; Jighly *et al.* 2016); for powdery mildew QTL on chromosomes 2B (Liu *et al.* 2001; Mingeot *et al.* 2002; Bougot *et al.* 2006; Bai *et al.* 2012), 2D (Bougot *et al.* 2006; Bai *et al.* 2012), 4A (Mingeot *et al.* 2002; Bougot *et al.* 2006; Chen *et al.* 2009), 5B (Bougot *et al.* 2006; Liu *et al.* 2017a), and 6D (Chen *et al.* 2009); and for Septoria QTL on chromosomes 1B (Chartrain *et al.* 2005; Tabib Ghaffary *et al.* 2011), 2D (Simón *et al.* 2004; Tabib Ghaffary *et al.* 2011), and 7B (Simón *et al.* 2004). However, an MTA identified for powdery mildew on the chromosome 3A has not been previously reported and it is potentially a novel MTA.

Area under disease progress curve (AUDPC) for leaf rust and stem rust was measured based on the repetitive disease severity assessments and is used to assess quantitative disease resistance in wheat accessions (Jeger and Viljanen-Rollinson 2001). Two MTAs identified for leaf rust AUDPC were also reported in earlier studies on chromosomes 1B (Schnurbusch *et al.* 2004) and 3B (Lu *et al.* 2017). However, six MTAs identified for leaf AUDPC on 1A, 1D, 5A, 6A, 6B, and 6D, and all seven MTAs for stem rust AUDPC have not been reported previously and are potentially novel MTAs controlling these traits.

### MTAs for grain quality traits

Several MTAs identified for grain quality traits such as grain protein and gluten content have been previously reported in the literature. For instance, the previous QTL similar to our study for grain protein concentration were on chromosomes 1A (Li *et al.* 2013), 1D (Pushpendra *et al.* 2007; Li *et al.* 2013), 2A (Pushpendra *et al.* 2007; Li *et al.* 2013), 2D (Pushpendra *et al.* 2007), 3A (Pushpendra *et al.* 2007), 3B (Sun *et al.* 2008a; Heo and Sherman 2013), 4A (Pushpendra *et al.* 2007; Li *et al.* 2013), and 5B (Heo and Sherman 2013). Similarly, QTL for gluten content on chromosomes 3B (Liu *et al.* 2016b) and 7D (Li *et al.* 2013) were identified previously. However, five MTAs identified for gluten content on the chromosomes 2A, 5A, and 5B have not been reported previously and are potentially novel MTAs responsible for gluten content in wheat.

### Putative candidate gene annotations

The variant effect predictor available on Ensemble was used to determine the functional consequences of the variant within 5 kb of the SNP location. In this study, 116 significant SNPs were identified within genes based on in silico candidate gene analysis. Of which, 21 SNPs were annotated as a missense variant and had moderate impact on genes. These missense variants cause an amino acid change and such changes may alter the function of genes, which makes these annotated gene as a strong candidate gene for future functional characterization studies in wheat. Some of the genes such as NBS-LRR disease resistance protein (Bhatta *et al.* 2018a, 2018b, 2018d), Protein DETOXIFICATION (Yokosho *et al.* 2016; Bhatta *et al.* 2018b), Zinc finger protein (Ma *et al.* 2009; Bhatta *et al.* 2018b), F-box protein (Lechner *et al.* 2006; Bhatta *et al.* 2018b), and Kinase family protein (Yan *et al.* 2017; Bhatta *et al.* 2018b) were also previously identified as important genes in wheat for agronomic and disease related traits (Bhatta *et al.* 2018a, 2018b, 2019a). We have identified MTAs associated with the same or associated traits located within genes whose annotation is exactly same. For example, harvest index, spike harvest index, number of plants, total root length, and grain perimeter were located within genes annotated as F-box family protein. This result indicated that these putative gene families may play an important in controlling these traits.

### Multi-trait MTAs and favorable alleles

Several multi-trait MTAs identified in this study were also positively associated with each other. This result suggested that multiple traits may have similar genetic basis and simultaneous improvement of multiple traits could be possible. Additionally, most of the co-located MTAs were present in genes. We have identified several favorable alleles that increases the agronomic performance, disease resistance, and quality traits in wheat accessions. Although the number of favorable alleles distribution in different breeding program did not significantly vary from each other, alleles present in each breeding program were different for different traits, which indicates the use of diverse accessions of wheat for different breeding objectives. Genomic fingerprinting for MTAs with favorable and unfavorable allele distribution in 143 diverse accessions could assist in building strategies while integrating the useful traits in an elite breeding lines. This study have recommended top performing five lines with higher number of favorable alleles for multiple traits, indicating the potential use of these lines in a breeding program. The favorable alleles identified for multiple traits in this study could be useful for pyramiding superior alleles in elite wheat germplasm upon validation in an independent population.

## Conclusions

The present study found considerable useful genetic variation for improving multiple traits and recommended five superior lines for directly use in a breeding program. A total of 243 genomic regions for improving agronomic performance, disease resistance, and grain quality related traits were identified. Of these, 120 favorable genomic regions were found to be novel MTAs, where large numbers were distributed on the D genome, indicating the usefulness of diverse lines in improving the D-genome diversity in elite wheat germplasm. Furthermore, this study identified co-located multi-trait markers within important gene families. The present study fingerprinted 143 diverse wheat lines for trait-associated markers and provided a detailed understanding of genetic architecture of agronomic, disease resistance and quality-related quantitative traits using diverse accessions of synthetic and bread wheat lines. However, further investigation on identified genomic regions could assist multi-trait marker-assisted breeding program.
